# Colorectal Cancer Screening: A Comprehensive Review to Recent Non-Invasive Methods

**Published:** 2017-07-01

**Authors:** Leila Hamzehzadeh, Meysam Yousefi, Seyed-Hamidollah Ghaffari

**Affiliations:** 1 Department of Medical Genetics, Faculty of Medicine, Mashhad University of Medical Sciences, Mashhad, Iran; 2 Hematology, Oncology and Stem Cell Transplantation Research Center, Shariati Hospital, Tehran University of Medical Sciences, Tehran, Iran

**Keywords:** Colorectal cancer (CRC), Chromosome instability (CIN), Microsatellite instability (MSI), The CpG- island methylator phenotype (CIMP), Fecal screening kit

## Abstract

Colorectal cancer (CRC) is one of the most common cancers worldwide and considered to be one of the hassles in medical communities. CRC develops from precancerous polyps in the colon or rectum and is preventable and curable by an early diagnosis and with the removal of premalignant polyps. In recent years, scientists have looked for inexpensive and safe ways to detect CRC in its earliest stages. Strong evidence shows that screening for CRC is a crucial way to reduce the incidence and mortality of this devastating disease. The main purpose for screening is to detect cancer or pre-cancer signs in all asymptomatic patients. In this review, we holistically introduce major pathways involved in the initiation and progression of colorectal tumorgenesis, which mainly includes chromosome instability (CIN), microsatellite instability (MSI), the CpG island methylator phenotype (CIMP), and we then will discuss different screening tests and especially the latest non-invasive fecal screening test kits for the detection of CRC.

## Introduction

 About a quarter of all deaths in countries with awesternized lifestyle are caused by cancer^1^. Colorectal cancer (CRC) isthethird most common canceramongmenandthe second among women, and it is the fourth leading cause of cancer-related mortality worldwide^2^.In 2017, there will be diagnosed 95,520 and 39,910 new cases of colon and rectal cancer in the US, respectively^3^.

In the last few decades, the incidence of CRC has been rapidly increased in Asia^4^.Approximately,5-6% of theWesternpopulationwillsuffer from CRCduringtheir lifetime^2^^,^^5^. Obesity, a diet low in fruit and vegetable, physical inactivity, smoking and a sedentary lifestyle are risk factors for CRC^6^^,^^8^. In this regard, lack of physical activity has shown to have a strong effect in the development of CRC by reducing the risk 25%^9^. Long-term treatment with aspirin, a low-fiber and Mediterranean diet may prevent colorectal cancer as well^7^^,^^10^^,^^11^. Colorectal cancer is classified into three major forms: sporadic, hereditary and familial. About 75-80% of CRCs are sporadic type in which somatic mutations are frequently found and arenot associated with family history^12^.Hereditary colorectal cancer, Lynch syndrome and Familial adenomatous polyposis (FAP)account for 10% of all cases of CRC^13^. Several studies estimated that approximately 25% of all CRC cases are familial and do not follow the classical Mendelian inheritance pattern^14^^,^^15^.Over the past few years, scientists have found that CRC is a heterogeneous cancer and hard to be diagnosed and treated through identification of its molecular and genetic characteristics^16^. The screening programs have declined the incidence rate of colorectal cancer by 4.3 percent per year among people 50 and above, but the CRC incidence rate increased by 1.8 percent per year among people under 50 ^17^^, ^^18^, necessitating consideration of ages for screenings. Table 1 shows the classification of CRC.

**Table 1:** Sporadic and inherited colorectal cancer (CRC)

**Inherited CRC** *		**Sporadic CRC** *	
**CIN**	**MSI**	**CIN**	**MSI**
FAP (1%); Germline APC (AD)	Lynch syndrome (2- 5%); Germline MMR (AD)	APC, Tp53, DCC and K- RAS LOH	Hypermethylations of MLH1; Mutation of BRAF
MUTYH (1%) Germline bi- allelic MUTYH (AR)			

* Colorectal cancer (CRC) can be sporadic or inherited. Most CRCs are sporadic and arise through the chromosomal instability (CIN) pathway, but about 15% of sporadic tumors arise through the microsatellite instability (MSI) pathway. Inherited cancers can be associated with both the CIN pathway (familial adenomatous polyposis [FAP] and MUTYH- associated polyposis) and MSI pathway (Lynch syndrome). Lynch syndrome accounts for 2-5% of all CRCs. AD: autosomal dominant; AR: autosomal recessive; LOH: loss of heterogeneity; MMR: mismatch repair

At least, four genetic and epigenetic mechanisms have been explained in CRC: 1) chromosomal instability (CIN); 2) microsatellite instability (MSI); 3) the CpG island methylator phenotype *(CIMP)* and 4) other mechanisms including inflammation and microRNAs. In this review, we will holistically explain the mentioned mechanisms and will bring up eminent methodologies for the current screenings and detection of CRC. 

**Genetic abnormalities implicated in the chromosomal instability pathway**

More than 80 somatic mutations have beenidentified in CRC by sequencing, only a few number of these mutations are significantly associated with CRC^19^. 

**WNT signaling components**

Initial genetic changein sporadic colon cancer and FAP (familial adenomatous polyposis) tumorgenesisisan activation of Wnt pathway and abnormalities in chromosome 5q. WNT ligands belong to a large family of proteins that play very important role in the development of normal cells. WNT binds to the membrane receptors and triggers signaling cascade which is involved in an important process of embryonic development and adult cell homeostasis such as cell differentiation, cell polarity, and cell death^13^. Wnt pathways are divided into two common categories: canonical (β-catenin dependent) and non-canonical (independent of β-catenin) Wnt signaling pathways^4^^,^^20^^,^^21^. About 90% of sporadic colon cancers carry mutations in the WNT pathway^22^. APC gene, a tumor suppressor gene, has 15 exons and is located on chromosome 15q. APC proteins bind to β-catenin and are main components in the destruction complex. The APC mutations cause a truncated product with an abnormal function^22^^,^^23^. Beta-catenin is normally found in the cell membrane, but in the absence of APC, itis usually accumulatedin the nucleus^24^. Germline mutations in the APC gene are responsible for familial adenomatous polyposis (FAP), however, somatic mutations in APC occur in 80% of sporadic colorectal tumors. A familial colorectal cancer syndrome such as FAP withan autosomal dominant inheritance ischaracterized by the development of hundreds or thousands of adenomas in the colon and rectum; the average age at FAP is 39 years^25^. Attenuated FAP (AFAP) is characterized by the presence of less than 100 adenomatous polyps; the germline mutations occur in 5′ and 3′ of the APC gene. MYH-associated polyposis (MAP) is caused by mutations in the mutY homolog (MYH) gene. MAP is inherited in an autosomal recessive manner, and thus individuals with MAP have biallelic MYH mutations.  These patients often have no family history of colon cancer or polyps in their parents (although siblings may be affected). MAP and AFAP are oftenphenotypically similar^26^. 

**Aneuploidy: 18q loss**

DCC, SMAD2 and SMAD4 genes are all located on 18q and the loss of an allele accounts for 60% of CRC, and it is associated with a poor prognosis in stage II and III of CRC^27^. DCC gene plays important roles in the regulation of cell adhesion and migration and stimulates cell death in the absence of its ligand (netrin-1). Smad proteins are transcription factors that are involved in the transforming growth factor β (TGF-β) signaling pathway^28^^,^^29^. A germline mutation of SMAD4 can cause juvenile polyposis syndrome (JPS) which is associated with CRC ^27^^,^^29^.

**K-RAS gene**

During the last decade, scientists have been greatly studied RAS pathways. RAS (Kirsten rat sarcoma viral oncogene homolog) has three isoforms: K-RAS, N-RAS and H-RAS. Mutations in the RAS family are common in different cancers. K-RAS, N-RAS and H-RAS mutations are detected in 25-30%, 8% and 3 % of all human cancers, respectively (24, 30, 31). Mitogen-activated protein kinases (MAPK) and phosphoinositide-3 kinase (PI3K) pathways arethe main cellular pathways which the RAS protein operates^32^. K-RAS gene, located on 12q, is a proto-oncogene that encodes a GTP-binding protein. When mutation occurs in K-RAS gene, it can cause a loss of inherent GTPase activity/ and thus it permanently activatesthedownstream RAS-RAF-MEK-ERK pathway^33^. Approximately 30-50% of CRCs are known to have mutation in the K-RAS gene which suggests that aberrant K-RAS protein has an important role in the formation of tumor^34^. More than 90% of the mutations in the K-RAS gene happen at codon 12 and 13 (35). Several studies have demonstrated that K-RAS mutations are associated with a poor prognosis in aggressive CRC and are predisposing factors for CRC metastasis to liver^36^^, ^^37^.

**Tp53 gene**

Tp53 gene is a tumor-suppressor gene with 12 exons and 11 introns which is located on chromosome 17p^38^. Its mutations are one of the main steps in colorectal carcinogenesis. About 80% of TP53 mutations are missense mutations. As a tumor suppressor, Tp53 has different roles including the ability to induce cell cycle arrest, DNA repair, senescence, and apoptosis^39^. Furthermore, it has a number of transcription independent cellular activities essential for the maintenance of genomic stability. TP53 mutation is observed in about half of all colorectal cancer cases^40^^,^^41^. 

**Microsatellite instability pathway**

Microsatellites are short repeat sequences scattered all over the human genome. Microsatellite instability (MSI) is caused by an inactivity of the DNA mismatch repair (MMR) system. At least,7 proteins of theMMR system have beenidentified: MSH2, MLH1, MLH3, MSH6, MSH3, PMS1, and PMS2^42^. Microsatellite instability (MSI) pathway represents about 15% of thesporadic CRC and > 95% of the Hereditary Non-Polyposis Colorectal Cancer (HNPCC) syndrome^43^. HNPCC is an autosomal dominant genetic disorderthat is characterized by an onset < 50 yearsold and also with other malignant tumors, including endometrial and ovarian cancer^44^.Germline mutations in one of the MMR components are occurred in the HNPCC syndrome. In 90% of HNPCCsyndrome, mutations are present in hMLH1 and hMSH2^45^. A 40%–60% increased risk of developing endometrial cancer is associated with a defective hMSH2 and with a mutation in hMLH1, which increase the risk of developing CRC by 50%–80% ^44^^, ^^46^.

**MSI-H, MSI-L and microsatellite stable**

Investigators in the International Workshop on Microsatellite Instability recommended a panel of five microsatellite loci for identification of MSI. The approved panel includes two mononucleotide (BAT25 and BAT26) and three dinucleotide microsatellites (D5S346, D2S123, and D17S250). MSI is categorized into three forms: MSI or MSI-high (MSI-H) is defined as MSI at ≥ 2 (40%) of the five specified sites, MSI-low (MSI-L) as MSI at one marker, and microsatellite stable (MSS) when no instability is demonstrated at the markers^47^. MSI-H tumors have fewer mutations in K-ras and p53. BRAF V600E mutations are frequently seen in sporadic MSI-H CRC with methylated hMLH1, but not in HNPCC^48^.

**The CpG-island methylatorphenotype pathway**

The second common pathway in sporadic CRCs is the CpG-Island Methylator Phenotype (CIMP) pathway. Epigenetic alterations cause changes in the gene expression or in the function without changing the DNA sequence of that particular gene^49^. DNA methylation occurs commonly at the 5′-CG-3′ (CpG) dinucleotides. In humans, epigenetic changes are mostly caused by DNA methylation or histone modifications. Five markers have been chosen to serve as markers for CIMP: CACNA1G, IGF2, NEUROG1, RUNX3, and SOCS1. Methylation of at least three markers is considered as CIMP positivity (50). The promoter hypermethylationcauses the loss of genes expression which is involved in the cell cycle regulation, apoptosis, angiogenesis, DNA repair, invasion and adhesion.The CIMP pathway accounts for approximately 20–30% of the sporadic cases of CRCs^51^. Based on the presence of MSI and CIMP, CRC is classified into five molecular subtypes^52^^, ^^53^as shown in Table 2.

**Table 2:** Five molecular classes of CRC based on the presence of MSI and CIMP.

	**Classification**	**Characterization**	**Origin**
1	CIMP high/MSI high	BRAF mutation; MLH1 methylation	Serrated adenomas
2	CIMP high/MSI low or microsatellite stable	BRAF mutation; methylation of multiple gene	Serrated adenomas
3	CIMP low/MSI low or microsatellite stable	hromosomal instability; K-ras mutation; MGMTmethylation	Tubular, tubulovillus/ serrated adenomas
4	CIMP negative/microsatellite stable	Chromosomal instability	Traditional adenoma
5	HNPCC	Germline mutations in the mismatch repair (MMR) genes	Not associated with sessile serrated adenomas (54).

**Other molecular mechanisms involved in CRC tumorgenesis**

**Inflammatory pathway**

One of the critical components in the CRC initiation and progression is chronic inflammation. Chan etal.examined the influence ofC-reactive protein (CRP), Interleukin-6 (IL-6) and soluble tumor necrosis factor receptor 2 (sTNFR-2, a TNF-α receptor superfamily member) in CRC ina cohort of 33,000 women. Their results demonstrated an increased risk of CRC in women with high levels of sTNFR-2, but no association with other two markers was found. Remarkably, women with high baseline levels of sTNFR-2 who took aspirin had a lower risk of developing CRC^55^^, ^^56^. Therefore, inflammation is an important contributor to colorectal carcinogenesis, and thusanti-inflammatory drugs have a protective effect on CRC^57^.

**MicroRNAs**

MicroRNAs (miRNAs) are a group of small non-coding RNAs which contain 18-24 nucleotides and regulate protein expression mostly by inhibiting mRNA translation of genes involved in cell differentiation, development, proliferation and apoptosis^58^.Recently, scientists have discovered that miRNAs are associated with CRC pathogenesis ^59^. For instance, it has been reported that miR-145 and miR-143 are usually downregulated in precancerous adenomas compared to normal tissue ^60^.Inseveral studies,scientists found a downregulation of miR-143 and -145 in stool samples of CRCs as compared to healthy controls ^61^^,^^62^. Another study showed miR-144 to be upregulated in stool samples of CRC patients. Sensitivity and specificity of CRC detection were 74% and 87%,respectively^63^. Therefore, it has been suggested that dysregulatedmiRNAs may be useful markers for early detection or follow-upof CRC patients^60^. Therefore, miRNAs have great promises for the detection of precancerous adenomas and are being broadly studied for screening purposes in CRC.

**Colorectal cancer screening**

In recent years scientists have investigated new methods for a rapid detection of CRC that are lessexpensive, non-invasive and also have an appropriate sensitivity and specificity.Since 1985, death rate from CRC has been reduced because of early detection, and much of the reduction was due to the screening of people aged 50 to 75. There are a variety of methods and tests for the detection of CRC such as colonoscopy, sigmoidoscopy, fecal occult blood test (FOBT), fecal immunochemical test (FIT), double contrast barium enema (DCBE) and computerized tomography (CT) scan^64^^,^^65^. According to the American Cancer Society,if CRC is diagnosed at an early stage, the survival rate is more than 90%. In recent years, improvements have been made with the stool DNA testing as non-invasive and inexpensive tests for the diagnosis of CRC^66^.

**CRC screening tests**

As mentioned above, five screening tests are traditionally used for colorectal cancer: fecal occult blood test (FOBT), sigmoidoscopy, colonoscopy, barium enema and digital rectal exam. Moreover, some new screening tests have recently been studied: stool DNA testing and computerized tomographic colonography (CTC). In the following section, we will introduce these tests and their advantages.

**Flexible sigmoidoscopy**

More than 80% of CRC patients will be diagnosed if the left colon and rectum are examined by sigmoidoscopy. The purpose of this inexpensive method is to identify adenomas in patients between the ages 55-65^67^^, ^^68^.

**Colonoscopy**

Colonoscopy is the gold standard method for CRC screening. Using colonoscopy, we are able to examine the entire colon and rectum, but this method has some disadvantages such as being expensive, needing a bowel preparation beforehand and having a risk for rupture ^69^.

**Barium enema**

Like colonoscopy, this method examines the entire colon and rectum. It is cheaper than colonoscopy. However,the intestine needs to be prepared beforehand and is an invasive procedure^70^. Moreover, the barium enema fails in detecting flat or < 5 mm lesions, and also polypectomy or biopsy is not possible (Table 3).

**Table 3:** Common methods for colorectal cancer screening

**Test**	**Advantages**	**Disadvantages**	**Repeat of test**	**Sensitivity**		**Specificity**
				**CRC**	**AA**	
Flexible sigmoidoscopy	-Relatively easy and safe -No need to prepare the small intestine -No need for sedation	-Only detects a third of the intestine -Small polyps (< 5 mm) may not be detected -Whole polyps cannot be removed -Risk of bowel perforation may cause discomfort in people -Slight risk of bleeding and infection -Colonoscopy should be done if abnormal reported	Every 5 year	~50% (95% distal only) (79)	~50% (95% distal only) (79)	92% (79)
Colonoscopy	-Entire colon to be detected - Polyps can be removed -It can also detect other diseases	-Small polyps may not be recognized -Negligible risk of bowel perforation -The bowel preparation is important -Expensive -Need to sedative -Slight risk of bleeding and infection	Every 10 years	95% (79)	95% (79)	90% (79)
Barium enema	-Usually the entire colon can detected -Relatively safe -No need to sedation	-Small polyps may not be recognized -The bowel preparation is required -Polyps cannot be removed during the test -Colonoscopy should be done if abnormal reported	Every 5 years	94.2 (80)	98-99 (81)	99.6 (81)
CT colonography (virtual colonoscopy)	-Relatively fast and safe -Entire colon to be detected -There is no need for sedation	-Small polyps may not be detected -Need to prepare the entire intestine -Polyps cannot be removed during the test -Colonoscopy should be done if abnormal reported	Every 5 years	96% (82)	94% (83)	86.4% (84)- 96.3% (83)
Fecal occult blood test (FOBT)	- inexpensive -No Need to prepare the colon -Can be done at home	-May not recognize much of polyps and other diseases -Possible false positive reports -Colonoscopy should be performed if abnormal observed	Every 1 year	70% (79) 24% 93	24% (79)	93% (79)
Fecal Immunochemi cal test (FIT)	-Inexpensive -No need to requires bowel preparation -No need to diet -Easily done at home	Similar to FOBT	Every 1 year	90.1% (78)	90.6% (78)	92.3% (78)
Stool DNA test	-No Need to prepare the intestine -There is no need to diet -Could be done at home	Similar to FOBT and FIT	Every 3 years	Mentioned in table 4		

**Computerized tomography**

Computerized tomography (CT) is a new method for screening. This method is cost-effective and affordable in 5 minutes with similar sensitivity to colonoscopy and barium enema^69^. However, there are significant limitations in CT: first,the colon should be thoroughly clean; secondly,polypectomy is not possible. In addition, flat lesions are missed^71^.

**FOBT**

This test detects occult blood (hemoglobin enzymatically) in the upper and lower digestive tract^72^. However, several studies have demonstrated its limited sensitivity for advanced adenomas (11%) and cancer (13%). FOBT is a non-invasive method and if a positive result is found, a colonoscopy is then recommended. However, approximately 13% to 42% of positive FOBT cases have negative colonoscopy^73^. A systematic review showed that the sensitivity of FOBT for CRC is 51% to 100%, while its specificity is 90% to 97% which will be higher if the test is repeated annually or biennial^74^^,^^75^. 

**Fecal immunochemical test**

Fecal immunochemical test (FIT) detects human globin with a specific antibody (76). The sensitivity and specificity of this test are higher than FOBT (77, 78). FIT Specificity for the prediction of colorectal cancer and adenoma is 90.1% and 90.6%,respectively, and the sensitivity of this method for neoplasms in the colorectal cancer and adenoma are 92.3% and 33.9%, respectively ^78^.

**Colosure™ test kit**

Recently, scientists discovered that the Vimentin gene is hypermethylated by 53-84% in colorectal cancer.Closure**™** test kit (Laboratory Corporation of America, http://www.labcorp.com) identifies methylation in the Vimentin gene. The exact performance of this epigenetic marker in detecting advanced adenomas is largely unknown. However, the Colosure kit is the only commercial kit that is available for screening clinical colorectal cancer in the US. The sensitivity and specificity of this kit for colon cancer is 72.5-83% and 53-86.9 %, respectively (Table 4). 

**Table 4:** Common stool test kits used for detection of CRC

**Detect**	**Location**	**Function**	**In CRC**	**Prevalence**	**Sensitivity**		**Specificity**
**Colosure™**							
Vimentin gene	10p13	Activated in mesenchymal cell; encodes a member of the intermediate filament family. The protein encoded by this gene is responsible for maintaining cytoplasm integrity, and stabilizing cytoskeletal interactions (106)	highly methylated (86)	53-84% (85)	72.5-83% (89)		53-86.9%(89)
**Cologuard®**							
NDRG4 Gene	16q21q22.3	Is a tumor suppressor gene and belongs to the NDRG gene family (109)	Methylation of NDRG4 promoter is a potential biomarker for the noninvasive detection of colorectal cancer in stool samples (Hyper methylated) (108)	The positive detection rate of methylated NDRG4 was 72.4% (107)	92.3% (for colon cancer); 42% (for large adenoma) (97- 98)		87%(97-98)
BMP3 Gene	4q21	Bone morphogenic protein 3 (BMP3) is a member of the transforming growth factor beta (TGFB) Superfamily of cytokines, which includes BMPs, activins, and TGFB isoforms (110)	BMP3 gene is commonly methylated in colorectal cancers and adenomas but rarely in normal epithelia (85)	Methylation of BMP3 was detected 66% of cancers and 74% of adenomas (85)			
KRAS Gene	12p12.1	KRAS is a guanosine triphosphate/guanosinediphosphate (GTP/GDP)-binding protein and is widely expressed in various human cells. As a GTPase protein, KRAS is involved in intracellular signal transduction and mainly responsible for EGFR-signaling activation (112)	mutations impair the intrinsic GTPase activity of therefore causing KRAS proteins to accumulate in the GTP- bound, active form (111)	40-45%(34)			
ACTB Gene	7p22	ACTB is considered as a housekeeping gene so that its expression is not usually affected by changing conditions; therefore it is widely used as internal control for quantification of gene/protein expression (113)					
**PreGen-Plus™ **							
APC Gene	5q21	A tumor suppressor gene that plays an important role in the Wnt signaling pathway, intercellular adhesion, cytoskeleton stabilisation, cell cycle regulation, and apoptosis (116)	Inactivating mutations of APC promote tumorgenesis by triggering unregulated transcription of oncogenes such as c-myc and cyclin D1 (115)	~80% of all human colon tumors (114)	94% (96)		52%(96)
KRAS	12p12.1						
p53	17p	P53 is a well-known tumor suppressor gene which encodes a phosphoprotein with the ability to bind to DNA as a transcriptional factor. Downstream targets of p53 are involved in cell cycle arrest allowing damaged cell to either repair itself or be targeted for programmed cell death (40)	Mutations in p53 are occurred in relatively late steps of development of colorectal tumors and are important determinant of progression from adenoma to malignant tumor (118)	70% of CRC. Mutations of the p53 gene are among the commonest genetic alterations in all cancers (117).			
BAT26	The BAT-26 is a locus located in one of the MSH2 introns and consists of a 26-repeat adenine tract (123)	A quasimonomorphic marker formed by a poly-A tract (121, 122).	The BAT-26 locus has been shown to be sensitive marker of MSI, which manifests as a shortening in the size of the respective mononucleotide repeat in tumor DNA (119, 120)	Graziele et.al showed the most frequent microsatellite amplification was BAT26 (100%) andlower D17S2720 (85.4%) (119)			
**PKM2 test**							
PKM2 Gene	15q22	This gene encodes a protein with pyruvate kinase activity that catalyzes the formation of pyruvate from (126)	Based on immunohistochemical studies, PKM2 is highly expressed in colon cancer (124, 125)		79 (103)		81% (103)

The Closure kit cannot replace colonoscopy; it is usually used as an alternative test or screening method for patients who are dissatisfied with other (invasive) methods of screenings^85^^-^^90^.

**Cologuard® test kit**

The Cologuard® test kit (registered trademark of Exact Sciences in the U.S) is the first kit which detects colon cancer based on DNA stool sample. This kit identifies colon cancer depending on the DNA markers and blood in stool. The detection system in this kit is based on the identification, amplification and detection of the methylated DNA targets (NDRG4 and BMP3), K-RAS point mutations, and ACTB (a reference gene for quantitative estimation of the total amount of human DNA in each sample) which is performed using the Quantitative Allele-specific Real-time Target and Signal Amplification (QuARTS™) technology. Occult blood in the stool sample is prepared and analyzed for fecal occult blood in a quantitative enzyme-linked immunosorbent assay (ELISA) that determines the concentration of hemoglobin in the sample^91^. Recent studies indicate that the sensitivity of the Cologuard kit for diagnosis of colon cancer and large adenomas is 92.3% and 42%,respectively, withspecificityof87%^97^^-^^98^.

 In comparison with FIT, the Cologuard kit’s sensitivity is almost two-fold greater (42% vs. 24%) inidentifying advanced adenoma^92^. However, this method cannot be a replacement for the diagnostic colonoscopy. The kit should be prescribed and is useful for men and women > 50 years and for anyaverage- risk individuals^91^. Any diet or bowel preparation is not required. In some studies, 13% of people (individuals without cancer or pre-cancer) showed a positive result using this kit,sothey were asked to take a colonoscopy^93^. The Cologuard kit was approved by the US Food and Drug Administration (FDA) in 2014 and screening is recommended every three years ^94^.

**PreGen-Plus** **™** **test kit**

PreGen-Plus™ test kit (Laboratory Corporation of America, conducted by EXACT Sciences, LabCorp) is an assay used for an early detection of colon cancer and for any moderate-riskindividuals. It uses a multitarget assay panel that contains 21 point mutations in K-ras, APC and p53 genes, a microsatellite instability marker (BAT-26) and a proprietary marker, the DNA Integrity Assay (95). Records show that the sensitivity and specificity are almost 94% and 52% for CRC,respectively^96^. PreGen-Plus™ has not been cleared by the FDA ^97^. 

**Pyruvate kinase type M2 (PKM2) test**

Pyruvate kinase (PK) is an enzyme which catalyzes the formation of pyruvate from phosphoenolpyruvate (PEP), the rate-limiting step in glycolytic cascade. There are four PK isoforms: L, R, M1, and M2, each expressed in specific tissues^98^. The M2 isoform is a splice variant of M1 and is highly expressed during the embryonic development and tumor formation. It has been demonstrated that tumor cells exclusively express PKM2^99^^,^^100^. Another study reported that the expression of PKM2 is elevated in CRC and is also related to later stages and lymph metastasis of CRC ^101^. PKM2 is an important enzyme in the metabolism of tumor cells and is a tumor marker in CRC. Therefore, ELISA-based measurement of PKM2 is a new test for the detection of CRC in stool samples^102^. Sensitivity and specificity of this test for a CRC detection is 79% and 81%,respectively. Moreover, positive and negative predictive values are about 74% and 86%,respectively^103^.The efficacy of this test is equal toFOBT. Li etal.haveshownthat PKM2 test cannot be used alone for the screening of CRC due to the relatively low specificity and low positive predictive value^104^^, ^^105^.

## CONCLUSION

 Over the past several years, different methods have been discovered for an early detection of CRC. Invasive methods will likely be replaced by fecal DNA tests in the future. MiRNAs are also promises for an early detection of CRC. However, other critical matters should also be considered, including cost-effectiveness, optimal testing intervals, and strategies for a follow-up evaluation of patients who have shown a positive result on a fecal DNA test.
